# Cognitive decline and risk of dementia in older adults after diagnosis of chronic obstructive pulmonary disease

**DOI:** 10.1038/s41533-023-00342-x

**Published:** 2023-05-13

**Authors:** Aldana Rosso, Tomas Månsson, Karl Egervall, Sölve Elmståhl, Marieclaire Overton

**Affiliations:** Division of Geriatric Medicine, Department of Clinical Sciences in Malmö, Lund University, Malmö, Sweden

**Keywords:** Chronic obstructive pulmonary disease, Epidemiology

## Abstract

Cognitive screening has been proposed for older adults diagnosed with chronic obstructive pulmonary disease (COPD). Therefore, we examined the change over time in cognitive function and the risk of incident dementia in older adults after COPD diagnosis. A sample of 3,982 participants from the population-based cohort study Good Aging in Skåne was followed for 19 years, and 317 incident COPD cases were identified. The cognitive domains of episodic memory, executive function, and language were assessed using neuropsychological tests. Mixed models for repeated measures and a Cox model were implemented. Participants performed, on average, worse over time on all neuropsychological tests after COPD diagnosis in comparison to those without COPD, although statistical significance differences were only observed for episodic memory and language. The groups had a comparable risk of developing dementia. In conclusion, our results indicate that cognitive screening in the early stages of COPD may be of limited clinical relevance.

## Introduction

Chronic obstructive pulmonary disease (COPD) is characterized by limitations of airflow and progressive inflammation of the airways. COPD is common among adults aged ≥65 years (global prevalence: 14.2%; 95% CI [11.8–18.0]^1^). Reduced respiratory function can lead to hypoxemia and hypercapnia, known to affect brain function negatively, increasing the risk of cognitive dysfunction^2^. A recent study suggested that also depression may play a role in cognitive decline in older adults diagnosed with COPD^3^. The association between cognitive impairment and COPD is dependent on the severity of COPD^2,4,5^ and coexisting comorbidities^5–7^. COPD patients typically also have other comorbidities, such as cardiovascular disease or diabetes mellitus, or a combination thereof, which may contribute to cognitive decline to a varying extent^5^. The prevalence of cognitive deficits in older adults diagnosed with COPD varies between 3% and 61%^4,5^. In comparison, the prevalence of mild cognitive impairment is reported to be between 6% and 25% for individuals between 60 and 84 years of age^8^. Cognitive domains, such as learning and memory, attention, and, to some extent, executive function and language, have been found to be impaired in COPD patients^2,6,9–11^. The impact of COPD on social cognition has been less studied^12^. It has been argued that cognitive status should be part of the initial respiratory assessment for all COPD patients^13^ or for older COPD patients with hypoxemia, increased inflammation, and coexisting comorbidities^5^. As impaired cognition could be a sign of dementia, it is important to assess to which degree COPD increases the risk of dementia. Prior research presents inconsistent findings on the association between COPD and dementia^4,11,14^. The aim of this study was to investigate how cognition and the risk of incident dementia are affected in older adults after COPD diagnosis in order to provide evidence regarding the relevancy of cognitive screening in this population.

## Results

### Baseline characteristics

Baseline characteristics are listed in Table 1. During follow-up, 317 incident COPD cases were observed. The mean age at COPD diagnosis was 74 years (SD: 8 years). The average time to COPD diagnosis was 6 years from the baseline visit (SD: 4 years).

**Table 1:** Baseline characteristics of the study participants.

Baseline characteristics		Will develop COPD (*n* = 317)	Will not develop COPD (*n* = 3665)	All participants (*n* = 3982)
Sex	Male	142 (44.8)	1657 (45.2)	1799 (45.2)
	Female	175 (55.2)	2008 (54.8)	2183 (54.8)
Alcohol	No consumption	54 (17.0)	597 (16.3)	651 (16.4)
	Low consumption	174 (54.9)	2026 (55.3)	2200 (55.2)
	Moderate to high consumption	77 (24.3)	883 (24.1)	960 (24.1)
	Missing (*n*, %)	12 (3.8)	159 (4.3)	171 (4.3)
Heart disease	No	228 (71.9)	2717 (74.1)	2945 (74.0)
	Yes	89 (28.1)	948 (25.9)	1037 (26.0)
Cardiovascular disease	No	288 (90.8)	3371 (92.0)	3659 (91.9)
	Yes	29 (9.2)	294 (8.0)	323 (8.1)
Diabetes	No	289 (91.2)	3373 (92.0)	3662 (92.0)
	Yes	28 (8.8)	292 (8.0)	320 (8.0)
Age (years)	Mean (sd) [min, max]	67.7 (8.4) [59.6,93.1]	68.3 (9.8) [59.2,94.2]	68.2 (9.7) [59.2,94.2]
Formal education (years)	Mean (sd) [min, max]	10.04 (3.4) [6.0,23.0]	10.8 (3.8) [1.0,30.0]	10.8 (3.8) [1.0,30.0]
	Missing (n, %)	10 (3.2)	125 (3.4)	135 (3.4)
Montgomery–Åsberg depression rating scale (score)	Mean (sd) [min, max]	3.42 (5.3) [0.0,29.0]	2.5 (4.2) [0.0,30.0]	2.6 (4.3) [0.0,30.0]
	Missing (n, %)	20 (6.3)	294 (8.0)	314 (7.9)
Immediate recall test (number recalled words)	Mean (sd) [min, max]	7.1 (2.4) [0.0,15.0]	7.2 (2.4) [0.0,15.0]	7.2 (2.4) [0.0,15.0]
	Missing (n, %)	24 (7.8)	318 (8.7)	344 (8.6)
Fluency test (number of mentioned animals)	Mean (sd) [min, max]	20.5 (6.3) [3.0,38.0]	21.2 (6.5) [0.0,50.0]	21.2 (6.5) [0.0,50.0]
	Missing (n, %)	19 (6.2)	306 (8.4)	326 (8.2)
Digit span test (span)	Mean (sd) [min, max]	4.2 (1.1) [2.0,7.0]	4.2 (1.2) [0.0,8.0]	4.2 (1.2) [0.0,8.0]
	Missing (n, %)	21 (6.8)	296 (8.1)	318 (8.0)
Mini-mental state examination (score)	Mean (sd) [min, max]	27.2 (2.1) [19.0,30.0]	27.3 (2.6) [0.0,30.0]	27.3 (2.6) [0.0,30.0]
	Missing (n, %)	18 (5.9)	213 (5.8)	228 (5.7)

*COPD* chronic obstructive pulmonary disease, *MADRS* Montgomery–Åsberg depression rating scale. Due to rounding to the first decimal place, the sum of percentages may not add to or exceed 100%.

### Test scores over time

As seen in Table 2, the decrease in test scores per year is more pronounced for COPD participants compared to non-COPD peers. The differences between the groups were statistically significant for episodic memory and language (see Table 2). Since the difference observed for the executive function was not statistically significant, differences in global cognition were not formally tested. For completeness, all *p*-values are reported, although the *p*-value for MMSE is only nominal. The estimated average decline per year for each neuropsychological test is shown in Table 3.

**Table 2:** Model coefficients related to COPD obtained using a mixed model for repeated measures.

Domain	Test	Estimate of change of score per year (95% CI) *p*-value, ref: No COPD					
		Primary analysis	Sensitivity 1	Sensitivity 2: wave 1	Sensitivity 2: wave 2	Sensitivity 2: wave 3	Sensitivity 3
Episodic Memory	Immediate recall test (number recalled words)	−0.04 (−0.07; −0.00) 0.042	−0.01 (−0.07; 0.05) 0.678	−0.04 (−0.07; −0.01) 0.024	−0.01 (−0.10; 0.08) 0.761	0.03 (−0.15; 0.20) 0.753	−0.02 (−0.09; −0.05) 0.640
	Number of visits	5535	2725	3637	1183	715	3151
Language	Fluency test (number of mentioned animals)	−0.09 (−0.18; −0.01) 0.027	−0.12 (−0.25; 0.04) 0.150	−0.13 (−0.18; −0.03) 0.008	0.04 (−0.17; 0.24) 0.727	−0.01 (−0.32; 0.31) 0.965	−0.72 (−0.33; 0.01) 0.035
	Number of visits	5593	2743	3734	1161	698	3167
Executive function	Digit span test (span)	−0.01 (−0.02; 0.01) 0.475	−0.01 (−0.04; 0.01) 0.336	0.00 (−0.02; 0.01) 0.640	0.00 (−0.05; 0.05) 0.921	−0.04 (−0.10; 0.02) 0.190	−0.01 (−0.04; 0.02) 0.540
	Number of visits	5512	2704	3648	1170	694	3174
Global cognitive status	Mini-mental state examination (score)	−0.07 (−0.14; 0.01) 0.077	−0.12 (−0.25; 0.01) 0.065	−0.05 (−0.13; 0.03) 0.263	−0.06 (−0.18; 0.06) 0.300	−0.19 (−0.49; 0.12) 0.229	−0.09 (−0.25; 0.05) 0.210
	Number of visits	5830	2827	3878	1213	739	3299

The outcome is the change in neuropsychological test scores per year. Sensitivity analysis 1 refers to the analysis performed using study spirometry measurements to define COPD. Sensitivity analysis 2 regards the stratification of the primary model into study waves. Sensitivity analysis 3 only includes the first follow-up visit for each subject. *COPD* chronic obstructive pulmonary disease.

**Table 3:** Estimation of change in neuropsychological test scores per year.

Domain	Test	Group	Estimated change in score per year					
			Primary Analysis	Sensitivity 1	Sensitivity 2: Wave 1	Sensitivity 2: Wave 2	Sensitivity 2: Wave 3	Sensitivity 3
Episodic Memory	Recalled words (number recalled words)	No COPD	−0.07 (−0.08; −0.06)	−0.07 (−0.09: −0.06)	−0.07 (−0.09; −0.06)	−0.06 (−0.08; −0.04)	−0.09 (−0.12; −0.05)	−0.05 (−0.07; −0.04)
		COPD	−0.11 (−0.14; −0.07)	−0.09 (−0.14; −0.03)	−0.11 (−0.15; −0.08)	−0.07 (−0.16; 0.01)	−0.06 (−0.23; 0.11)	−0.07 (−0.14; −0.00)
		Number of visits	5535	2725	3637	1183	715	3151
Language	Verbal fluency test (number of mentioned animals)	No COPD	−0.25 (−0.28; −0.22)	−0.26 (−0.30; −0.22)	−0.23 (−0.26; −0.19)	−0.26 (−0.32; −0.20)	−0.29 (−0.38; −0.20)	−0.19 (−0.23; −0.16)
		COPD	−0.34 (−0.42; −0.26)	−0.36 (−0.50; −0.22)	−0.36 (−0.45; −0.27)	−0.22 (−0.42; −0.03)	−0.30 (−0.59; 0.00)	−0.36 (−0.52; −0.21)
		Number of visits	5593	2743	3734	1161	698	3167
Executive functioning	Digit span backward (size of the span)	No COPD	−0.02 (−0.03; −0.01)	−0.03 (−0.03; −0.02)	−0.02 (−0.03; −0.01)	−0.02 (−0.03; −0.01)	−0.01 (−0.02; 0.01)	−0.01 (−0.02; −0.01)
		COPD	−0.03 (−0.04; −0.01)	−0.04 (−0.07; −0.01)	−0.02 (−0.04; −0.01)	−0.02 (−0.07; 0.03)	−0.05 (−0.10; 0.01)	−0.02 (−0.06: 0.01)
		Number of visits	5512	2704	3648	1170	694	3174
Global cognitive status	Mini-mental state examination (score)	No COPD	−0.09 (−0.11; −0.07)	−0.07 (−0.09; −0.04)	−0.13 (−0.15; −0.11)	0.00 (−0.02; 0.02)	−0.03 (−0.09; 0.03)	−0.05 (−0.07; −0.03)
		COPD	−0.16 (−0.23; −0.08)	−0.19 (−0.31; −0.06)	−0.18 (−0.26; −0.10)	−0.06 (−0.18; 0.05)	−0.22 (−0.50; 0.06)	−0.14 (−0.29; 0.01)
		Number of its	5830	2827	3878	1213	739	3299

The estimates were obtained using a mixed model for repeated measures. Sensitivity analysis 1 refers to the analysis performed using study spirometry measurements to define COPD. Sensitivity analysis 2 regards to the stratification of the primary model into study waves. Sensitivity analysis 3 only includes the first follow-up visit for each subject. *COPD* chronic obstructive pulmonary disease.

### Dementia over time

Overall, 266 participants developed dementia during follow-up. Of those, 24 participants were diagnosed with COPD. The mean age of the participant when diagnosed with dementia was 84 years (SD: 7 years). The estimated incidence of dementia was 7.6 per 1000 person/years (95% CI [6.7; 8.5] *p*-value < 0.001). The hazard ratio of developing dementia between COPD and non-COPD participants was 1.2 (95% CI [0.8; 1.9], *p*-value = 0.319). The performed sensitivity analysis using spirometry measurements to define COPD yielded similar results (HR = 1.1, 95% CI [0.6; 2.0], *p*-value = 0.659).

## Discussion

Previous studies indicate that older adults diagnosed with COPD are at higher risk of cognitive decline compared to peers without this diagnosis^13–15^. COPD patients with cognitive impairment have poor compliance with medication and oxygen therapy, which increases the risk of acute exacerbations^9^. Since cognitive screening has been proposed for this population, further understanding of cognitive decline in COPD is necessary. This study examined how different cognitive domains were affected over time in older adults after being diagnosed with COPD. The COPD participants received their diagnosis on average 6 years into the study, and most of the patients were still at an early stage of the disease. On average, participants performed worse over time after COPD diagnosis in all tested cognitive domains, that is, episodic memory, executive function, and language. The differences between COPD and non-COPD participants were statistically significant only for episodic memory and language. However, all outcomes of this study point in the same direction, suggesting that early-stage COPD could affect cognition in a negative way, which is concordant with what has been reported previously^14,16–20^. One previous study provides evidence that COPD is not associated with cognition but that depression may instead contribute to the observed cognitive decline in older adults^3^. In our study, we observed a worsening of COPD despite statistical adjustments for depressive symptoms. The impact of early COPD on cognition seems however limited since the differences in test scores between the groups were minor and, therefore, suggestively irrelevant clinically.

To contextualize the estimated statistically significant annual change in scores for the recalled word test and the animal fluency test, we compared our results to those previously observed in older adults with cognitive deficits. A recent clinical study^21^ implemented the HVLT-R test, which is a similar test to our word recall test. The authors considered a decrease of 6 or more words at 24 months to be an indication of cognitive decline^21^. We estimated that, on average, participants without COPD would recall 0.07 words less per year (Table 3), yielding a decrease of 0.14 words after 24 months. For the group of participants with COPD, the change after 24 months was estimated to be 0.22 fewer words. For both groups, the average change is less than 1 word per 24 months. The annual change in the result of the animal fluency test in cognitively healthy older adults has been estimated to be approximately −0.24 animals, compared to −0.69 animals in patients with preclinical Alzheimer’s disease^22^. We estimated an annual result change of −0.25 and −0.34 animals in non-COPD and COPD participants, respectively. The annual change in both groups is much smaller than that observed for older adults diagnosed with preclinical dementia. The estimated annual change in both the recalled word test and the animal fluency test is much smaller than those reported for older adults with cognitive deficits. Thus, we suggest that these changes are not clinically relevant. In addition, participants recently diagnosed with COPD have stable MMSE scores, indicating that the impact of early COPD on global cognition is minimal. This is concordant with our previous finding that accelerated decline in lung function is limited at early-stage COPD^23^.

The mechanism behind a potential accelerated cognitive decline in COPD remains unclear^15^. This work provides support for the hypothesis that arterial hypoxia and hypercapnia could be two of the main drivers of cognitive decline in COPD since cognitive deterioration, besides the normal decline observed in aging, seems to be limited in early-stage COPD patients. Of note, more than 80% of older patients with COPD are also diagnosed with other comorbidities such as cardiovascular disease and diabetes^24^, also affecting cognition. Therefore, the potential contribution of early-stage COPD on cognitive decline is entangled with that of other comorbidities and unhealthy lifestyle factors, such as smoking.

Patients with COPD could be at increased risk of cognitive impairment and subsequent dementia^4^. We estimated a statistically non-significant, slightly higher risk of developing dementia for COPD participants. A recent study found the prevalence and incidence of dementia in COPD patients to be similar to that of age-matched controls^4^. The interpretation of these findings is not straightforward. Firstly, COPD patients are at higher risk of death due to COPD itself and common COPD comorbidities such as cardiovascular disease. Secondly, mild COPD and mild dementia can be difficult to diagnose in older adults since cognition and lung function normally declines with aging. Thirdly, it has been suggested that the pattern of cognitive impairment in COPD patients may differ from that observed in Alzheimer’s disease^10^. This may imply that COPD increases the risk for non-AD types of dementia, rendering it problematic to find an association between dementia and COPD, at least when examining all-cause dementia. Thus, we believe that an association between COPD and dementia may be plausible, but it is probably blurred by higher mortality in COPD patients as well as difficulties in establishing the diagnosis of dementia and COPD in older adults.

Current empirical evidence suggests that screening for cognitive impairment does not necessarily improve decision-making or important patient, caregiver, or societal outcomes in community-dwelling older adults^25^. For COPD patients, it has been argued that cognitive screening should be part of the initial respiratory assessment for all patients^13^ or for older COPD patients with hypoxemia, increased inflammation, and coexisting comorbidities^5^, as cognitive impairment is highly prevalent in this group. However, a recent review^15^ could not establish enough empirical evidence that COPD causes cognitive impairment. Our study shows that recently diagnosed COPD older adults, on average, perform only slightly worse on cognitive tests compared to adults without COPD. Therefore, we argue that cognitive screening of recently diagnosed older adults could be redundant. Nevertheless, in case patients show an indication of cognitive decline, their cognitive status should be assessed regardless of the COPD stage.

Cognitive decline in older adults diagnosed with COPD followed up to 19 years was investigated. Despite including 12 neuropsychological tests, instruments measuring social cognition were lacking. In addition, episodic memory was assessed by immediate word list recall but not delayed recall. A delayed recall measure was available from a separate five-item test but was not included in this study because of ceiling effects in the healthy older population. It is worth noticing that performance on an immediate recall test alone, although clinically informative, probably cannot be equated with episodic memory^26^. The impact of COPD on social cognition is poorly understood^12^, and future investigation is warranted. We acknowledge that the interpretation of our results is limited by the lower numbers of dementia and COPD cases. Participants in the GÅS study should be both willing and capable of completing an approximately 4-h-long cognitive and physical examination. Frail individuals interviewed at nursing homes mostly performed a limited examination. Illiterate older adults are not represented in our study sample. Therefore, literate, healthy, and overall cognitively intact participants are over-represented in the study sample, and our conclusions may not be generalizable to other groups. Participants with advanced COPD may not be capable of attending the study visits due to their deteriorated health and are, therefore, likely underrepresented in the study. The mentioned effects would reduce the difference in test scores between COPD and non-COPD groups. The median follow-up time during the study was 7 years, and therefore long-time effects in cognitive deterioration could not be observed. Further work is required to understand whether cognitive screening would be helpful for advanced COPD patients. Although COPD was clinically diagnosed and dementia was defined using standardized procedures complemented with health-care records, there is a risk of misclassification. For example, some COPD participants may have received their diagnosis in a delayed manner since the COPD symptoms may have been attributed to other diseases, in particular in non-smokers. We performed a sensitivity analysis using study spirometry measurements to identify COPD participants. The results of the sensitivity analyses were largely concordant with those presented in the primary analysis. Therefore, we believe that the potential impact of COPD misclassification on the results is minor. We also investigated potential cohort effects and the impact of attrition in sensitivity analyses. Similar results as in the primary analysis were rendered, which alleviates our concern. Despite collecting extensive information about health status and lifestyle, unmeasured confounding remains.

In conclusion, this study presents indications that additional cognitive decline due to COPD is seemingly minor at an early stage of the disease. Therefore, we conclude that cognitive screening of patients at an early stage of COPD is most likely unnecessary. The cognitive status of patients with indications of cognitive decline should be investigated regardless of the COPD stage.

## Methods

### Study design and participants

**Fig. 1:**
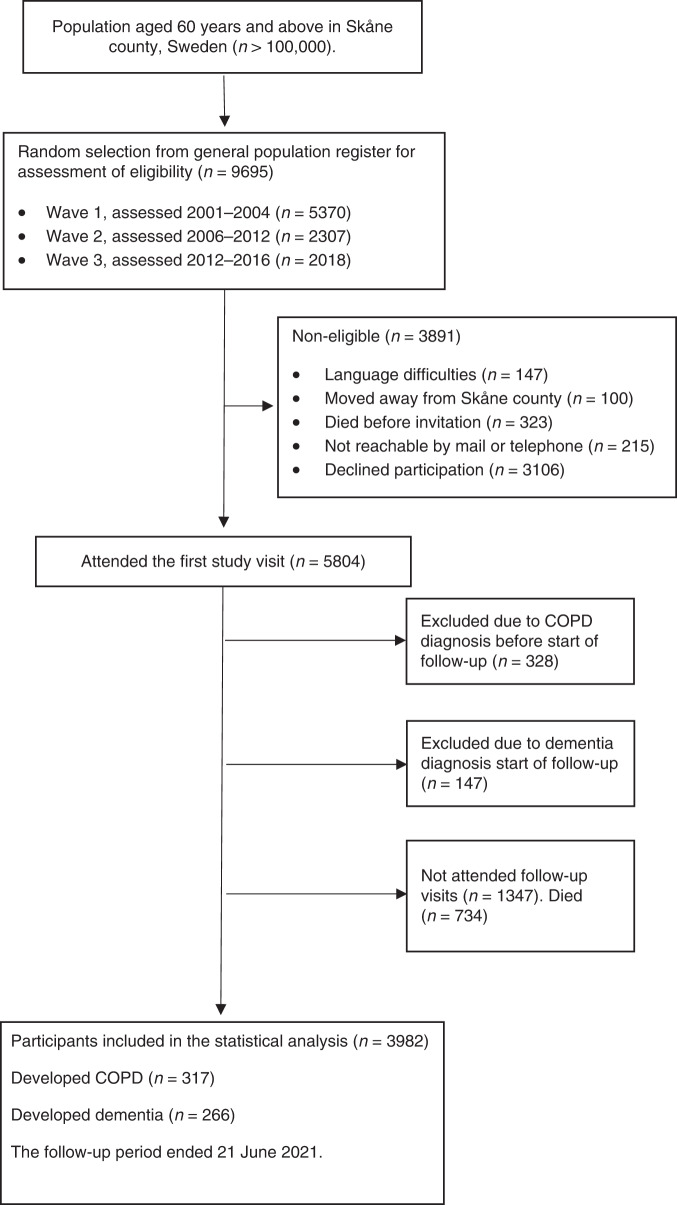
Study population. This flowchart illustrates how the study population was selected.

The Good Aging in Skåne (GÅS) is a prospective, longitudinal population-based study aiming to investigate different aspects of aging^27^. Briefly, 60- to 93-year-old subjects living in the south of Sweden, Skåne, are randomly selected from the Swedish population register. Participants are offered a thorough physical, medical, and psychological examination and are invited to follow-up examinations at regular intervals every three (≥78 years of age) to six (<78 years of age) years until death. To encourage the participation of frail adults, the study team performs home visits following the same study protocol. However, patients living at nursing homes or with limited mobility may receive a shorter examination if they are not able or willing to perform a full examination. Currently, three waves have been fully recruited, with an initial participation rate of approximately 60%. As shown in Fig. 1, data from 5804 participants examined between 13th Feb 2001 and 21st Jun 2021 were retrieved. In order to study early-onset progression and to minimize bias, participants diagnosed with COPD and/or dementia at the first study visit were excluded from this analysis. The GÅS study is conducted according to the Declaration of Helsinki and Good Clinical Practice guidelines. The study is approved by the Lund University Ethics Review Board (LU 744-00). All participants provided written informed consent.

### Cognitive assessment

Cognition in the GÅS study was assessed using a neuropsychological test battery including 12 instruments administrated by experienced test administrators following a standardized protocol. In this work, we focused only on cognitive functions that were most frequently found to be affected in COPD patients in previous studies (global cognition, memory, executive function, and word fluency^2,10,14,16,18,20,28^). Global cognitive status was assessed using the mini-mental state examination (MMSE). Episodic memory was assessed using a 16-word free recall test. Executive function was assessed using a modified version of the WAIS-III digit span backward test. The language domain was assessed using a categorical verbal fluency test (animals). A description of the GÅS test battery has been presented elsewhere^29^.

### Socio-demographics and morbidities

Socio-demographic variables included age, sex, and years of formal education. Alcohol consumption was self-reported. Morbidities were identified by several methods: self-reports, medical examination, reviewing medical records, and linking to the Skåne Healthcare Registry (SHR). In the SHR, diseases are classified according to the International Classification of Diseases System version 10 (ICD-10). Cerebrovascular disease included cerebral infarction, cerebral hemorrhage, occlusion and stenosis of precerebral or cerebral arteries, and/or transient cerebral ischemia. Heart disease included acute myocardial infarction, ischemic heart disease, presence of cardiac and vascular implants, heart failure, and atrial fibrillation or other arrhythmias. COPD also includes emphysema and chronic bronchitis. During the study visit, symptoms of depression were assessed using the Montgomery–Åsberg depression rating scale (MADRS). Dementia diagnosis was primarily extracted from medical records and included Alzheimer’s disease, mixed dementia, vascular dementia, Lewy body dementia, and frontotemporal dementia. Complementary diagnosis of all-cause dementia was classified by the study physician during thorough examination according to the Diagnostic and Statistical Manual of Mental Disorders 4th edition (DSM-IV). The date of death was obtained by linkage to the Swedish people and address register.

### COPD diagnosis

Participants with COPD were diagnosed in standard clinical practice. Clinically, diagnosis of COPD in Skåne county is based on three criteria: spirometry verified obstructive after bronchodilation, current airway symptoms, and a history of risk factors for COPD.

### Spirometry assessments

During the study visits, a spirometry assessment was performed using a Vitalograph 2120 spirometer (Vitalograph Ltd., Buckingham, UK) according to the American Thoracic Society guidelines^30^. Bronchodilators were not administrated during the first wave baseline visit. Subjects received 1.0 mg of β2-receptor agonist terbutaline 10 min prior to the spirometry in the majority of visits. The spirometry results were used in a sensitivity analysis as an alternative way to identify COPD cases.

### Statistical analyses

Mixed models for repeated measures were implemented to estimate the mean neuropsychological test scores over time. The models had a random intercept for each participant. The rate of change of neuropsychological test score was calculated as the difference between the score at the follow-up visits and the baseline score, divided by the time between the visits. Using a directed acyclic graph, we identified which factors were required in the model to estimate the direct effect of COPD on cognitive performance. Age, sex, education, alcohol consumption, heart disease, cerebrovascular disease, depression score, and diabetes at baseline were then included to control for confounding. Since our model is optimized for the interpretation of the coefficients related to COPD, no conclusion is drawn regarding the effect of the other covariates on cognition^31^. In order to improve precision, we also included the test score at baseline in the models. Confidence intervals were calculated using robust standard errors. The goodness of the fit was assessed visually using residual plots. In this study, there were several statistical hypotheses of interest (one for each cognitive domain). To reduce the burden of multiplicity, we implemented a fixed sequence testing procedure^32^. Briefly, we ordered the cognitive domain hypotheses according to our a priori expectations of observing a difference between COPD and non-COPD participants. In this procedure, the first hypothesis is tested, and if rejected, the second is tested. The testing procedure continues until a hypothesis cannot be rejected. This hierarchical testing approach controls the type I error at 5% in the strong sense. The implemented testing order was (1) immediate recall test, (2) fluency test, (3) digit span test, and (4) MMSE. An extended Cox model using baseline characteristics (age, sex, education, heart disease, cerebrovascular disease, and diabetes) was implemented to estimate the risk of dementia for participants with and without COPD. The presence of COPD diagnosis was modeled as a time-dependent covariate. Violations of the proportional hazard assumption were assessed visually using log-log plots, as well as with a formal test, in order to investigate whether the log hazard-ratio function was constant over time. The overall prevalence of dementia and COPD in the GÅS study are 6.5%^33^ and 5.5%^23^, respectively. Therefore, the number of incident dementia cases in COPD participants in this study was expected to be low. Dementia is thus presented as a secondary outcome and excluded from the testing hierarchy.

### Sensitivity analyses

Potential sources of bias were COPD misclassification, cohort effects, and attrition. Assigning a correct COPD diagnosis in older adults with comorbidities is difficult, mainly due to overlapping symptoms and difficulties performing a correct spirometry^34^. To overcome the uncertainty in COPD diagnosis in clinical practice, sensitivity analyses were performed using spirometry measurements collected during the study visits to identify COPD cases. Participants whose ratio of forced expiratory volume in the first second (FEV1) and forced vital capacity (FVC) was <0.7 were classified as having COPD. The data were collected over the course of 20 years, and therefore cohort effects are expected. The primary models were stratified by study wave to investigate this issue. Attrition is inevitable in cohort studies involving older adults. Thus, we conducted sensitivity analyses considering only the first follow-up visit. The statistical analyses were performed using Stata IC 17.0 software (StataCorp LLC, TX, USA) and Python 3.8.5 (Python Software Foundation).

### Reporting summary

Further information on research design is available in the Nature Research Reporting Summary linked to this article.

## Supplementary information

Reporting Summary

## Data Availability

Data are accessible on request (https://neardb.near-aging.se/study/gas-snac-s).
